# Validity of aerobic capacity indicators derived from the progressive specific taekwondo test for prescribing sport-specific interval training

**DOI:** 10.3389/fphys.2025.1572605

**Published:** 2025-07-11

**Authors:** Gennaro Apollaro, Marco Panascì, Ibrahim Ouergui, Emerson Franchini, Piero Ruggeri, Coral Falcó, Emanuela Faelli

**Affiliations:** ^1^ Department of Neuroscience, Rehabilitation, Ophthalmology, Genetics and Maternal Child Health, University of Genoa, Genoa, Italy; ^2^ Centro Polifunzionale di Scienze Motorie, University of Genoa, Genoa, Italy; ^3^ Department of Experimental Medicine, Section of Human Physiology, University of Genoa, Genoa, Italy; ^4^ High Institute of Sport and Physical Education of Kef, University of Jendouba, Jendouba, Tunisia; ^5^ Research Unit, Sports Science, Health and Movement, University of Jendouba, Jendouba, Tunisia; ^6^ Martial Arts and Combat Sports Research Group, Sport Department, School of Physical Education and Sport, University of São Paulo, São Paulo, Brazil; ^7^ Department of Sport, Food and Natural Sciences, Western Norway University of Applied Sciences, Bergen, Norway

**Keywords:** aerobic training, combat sports, endurance assessment, heart rate deflection point, mobile technology

## Abstract

**Background:**

The Progressive Specific Taekwondo Test (PSTT) is used to determine indicators of aerobic capacity (heart rate deflection point [HR_DP_] and kick frequency at the HR_DP_ [KF_DP_]) useful for prescribing sport-specific training. The aim of this study was to prescribe a sport-specific interval training (IT) session at the KF_DP_, identified during the PSTT, and to investigate HR response and muscle power performance.

**Methods:**

Thirteen taekwondo athletes of national and international level (mean ± SD: age: 17.6 ± 2.7 years) voluntarily participated in the study. In two experimental sessions, athletes performed: the PSTT to identify HR_DP_ and KF_DP_; a sport-specific IT at the KF_DP_, using the technical action of bandal-chagi (3 rounds × 2 min/1 min of recovery in-between). During each round of the IT, the HR was recorded to identify the HR_ROUND_. Before and after each round of the IT, muscle power performance was assessed through three countermovement jump (CMJ) tests.

**Results:**

HR_ROUND_ was significantly correlated with HR_DP_ (*r* = 0.774–0.789; *p* < 0.01). During round 1, HR_ROUND_ was significantly lower (*p* = 0.008) than HR_DP_. In rounds 2 and 3, there were no difference (*p* = 0.067 and *p* = 0.653, respectively) between HR_ROUND_ and HR_DP_. No difference was observed between pre- and post-IT CMJ performance (*p* = 0.210). Post-IT CMJ performance was significantly greater (*p* = 0.009) than that in the interval between rounds 1 and 2.

**Conclusion:**

During the IT rounds, expected HR responses emerged and muscle power performance was not compromised. Thus, these results support the use of PSTT-derived HR_DP_ and KF_DP_ as practical, sport-specific indicators for tailoring IT intensity in taekwondo athletes.

## 1 Introduction

In the last decade, research has focused on the development and use of sport-specific tests for assessing endurance in taekwondo athletes, in parallel with the widespread use of general tests (Bridge et al., 2014; Hausen et al., 2018; Sant’Ana et al., 2019; Araujo et al., 2021; Taati et al., 2022; Apollaro et al., 2024a). A recent review reported that the currently available sport-specific protocols for endurance are simple to conduct, noninvasive, and easily accessible to stakeholders (Apollaro et al., 2024a). On the other hand, each test has specific methodological and application characteristics, particularly the time structure and aerobic indicators derived, that must be appropriately considered when choosing which test to use (Hausen et al., 2018; Sant’Ana et al., 2019; Araujo et al., 2021; Taati et al., 2022; Apollaro et al., 2024a). To date, the Progressive Specific Taekwondo Test (PSTT) is the most studied test and it has a time structure that was defined following the recommended criteria to identify the heart rate deflection point (HR_DP_) in maximal progressive tests (Conconi et al., 1996). The concurrent criterion validity of the PSTT was established with the incremental treadmill test (ITT) (Sant’Ana et al., 2019) and the multistage shuttle run test (SRT) (Rocha et al., 2016). In addition, its test-retest reliability was investigated (Sant’Ana et al., 2017; Ouergui et al., 2020; Ouergui et al., 2021). Sant’Ana et al. (2019) found no significant difference for maximal oxygen consumption (VO_2MAX_), oxygen consumption at HR_DP_ (VO_2HRDP_), and HR_DP_ between the PSTT and ITT. In particular, the similar HR_DP_ between the two tests supported the assumption that the PSTT can provide indicators of exercise intensity, based on HR, easily applicable to monitor not only power but also aerobic capacity in a sport-specific mode (Sant’Ana et al., 2019).

It is important to highlight that the PSTT was originally developed to indirectly estimate the anaerobic threshold (AT) from the HR_DP_ (Sant’Ana et al., 2009). Sant’Ana et al. (2009) found that HR_DP_ and kick frequency at HR_DP_ (KF_DP_) in the PSTT were not different and significantly correlated with heart rate at AT (HR_AT_) and kick frequency at AT (KF_AT_) during a sport-specific constant load test at the fixed concentration of 4 mmol^.^L^-1^ of blood lactate [La]. Thus, the PSTT is the only test among those available that allows the determination of both aerobic capacity (i.e., HR_DP_ and KF_DP_) and power indicators (i.e., maximal heart rate [HR_MAX_] and maximal kick frequency [KF_MAX_]) useful for subsequent sport-specific training’s prescription (Apollaro et al., 2024a). In contrast, the validity of the fundamental aerobic variables of other sport-specific tests (such as the Continuous Taekwondo-Specific test, Interval Taekwondo-Specific test, Continuous Taekwondo-Specific Cardiopulmonary test, and Interval Taekwondo-Specific Cardiopulmonary test) (Hausen et al., 2018; Araujo et al., 2021) was either not confirmed or no aerobic variables (as in the Taekwondo-Specific Aerobic-Anaerobic-Agility test) were provided for monitoring and prescribing training (Taati et al., 2022). Sant’Ana et al. (2017) determined aerobic power indicators (i.e., HR_MAX_ and KF_MAX_), during the execution of the PSTT, aiming to investigate the acute effects of a sport-specific time-to-exhaustion test performed at the previously found KF_MAX_. Until then, this study was the only one that prescribed sport-specific training from indicators derived from PSTT in taekwondo athletes. Aiming to facilitate and increase the applicability of this testing protocol, the *ITStriker* application (*ETS4ME, São José, SC, Brazil*) was developed. This mobile technology reproduces the typical time regression and linear increase of KF of the PSTT, and automatically derives the internal and external load parameters for their subsequent use in “training mode” (Antonietto et al., 2021; Sant’Ana et al., 2021; Rodrigues et al., 2023; De Oliveira et al., 2024). To date, the rapid real-time data processing and low cost of this app facilitated the execution of the PSTT (Antonietto et al., 2021), the adaptation of the protocol to other combat sports such as karate (Rodrigues et al., 2023) and boxing (De Oliveira et al., 2024), and the first attempt of sport-specific training prescription for taekwondo (Sant’Ana et al., 2021).

Specifically, Sant’Ana et al. (2021) determined indicators of aerobic capacity (i.e., HR_DP_ and KF_DP_), while performing the PSTT and using the *ITStriker* app. Their objective was to analyze the acute effects of a sport-specific interval training (IT) session that mimics the time structure of the official match (i.e., 3 rounds × 2 min/1 min of recovery in-between), performed at the previously found KF_DP_ and reproduced by the *ITStriker* app, on HR responses, countermovement jump (CMJ) test performance, and parameters associated with neuromuscular and motor performance of the bandal-chagi (i.e., roundhouse kick) (Sant’Ana et al., 2021). Among the results, it was reported that only HR response during round 1 of IT was significantly lower than that observed at the same intensity (i.e., KF_DP_) during the PSTT. In parallel, CMJ performance (measured before and after each round of the IT) showed no differences between the pre- and post-IT (Sant’Ana et al., 2021). These results supported the use of HR_DP_ and KF_DP_, obtained during the PSTT, to prescribe IT as expected HR responses emerged and muscle power performance was not compromised (Sant’Ana et al., 2021). However, focusing on the IT protocol, athletes were allowed to perform kicks with technical variations and combining up to three kicks for each sound signal (Sant’Ana et al., 2021). Although this was conducted to reproduce the technical-tactical actions closest to those performed in combat (Sant’Ana et al., 2021), during the PSTT the athlete performs only one bandal-chagi for each sound signal (Sant’Ana et al., 2021). Thus, the different and specific technical-tactical actions of IT might have altered the KF required from the athlete generating HR responses and muscle power performance intensity-specific.

In this sense, to support in the first instance the validity of the aerobic capacity indicators derived in the PSTT (i.e., HR_DP_ and KF_DP_), to prescribe sport-specific training, it is be appropriate to structure an IT that includes the single technical action of the test (i.e., bandal-chagi) for each sound signal. This step would incentivize the use of aerobic capacity indicators derived from the PSTT to tailor IT intensity. Therefore, the aim of this study was to prescribe a sport-specific IT session (which mimics the time structure of the official match), at the KF_DP_ identified during the PSTT, using the technical action of bandal-chagi, and to investigate HR response and muscle power performance, through CMJ tests, in taekwondo athletes. We hypothesized that: (a) the HR during the three rounds of the sport-specific IT at the KF_DP_ would be lower only in the first round, compared with that observed at the KF_DP_ during PSTT, due to the kinetics of HR as a result of the specific high-intensity required. Consequently, the HR would be similar in the second and third rounds, compared with that observed at the KF_DP_ during PSTT, as HR_DP_ is generally identified at a percentage of HR_MAX_ located in the heavy domain of exercise (Ribeiro et al., 1985; Bunc et al., 1995; Bodner and Rhodes, 2000); (b) three rounds of the sport-specific IT at the KF_DP_ would not cause a decrease in muscle power performance. The CMJ test is commonly used to assess lower limb muscle power in taekwondo athletes, and its positive influence on the ability to repeat high-intensity intermittent efforts is documented (Albuquerque et al., 2022; Apollaro et al., 2024c). In this sense, the hypothesis that the KF_DP_ does not impair muscle power performance would indicate the integrity of the mechanisms underlying the neuromuscular system during the repeated bandal-chagi technical action.

## 2 Materials and methods

### 2.1 Study design

Athletes conducted two distinct experimental sessions. On the first day, anthropometric and body composition measurements (i.e., body height, body mass, and body fat) were performed as well as the PSTT to identify HR_DP_ and HR_MAX_, and their associated KFs (KF_DP_ and KF_MAX_, respectively). On the second day, after a 48-h interval, athletes performed a sport-specific IT session at the KF_DP_ identified during the PSTT. Before and after each round of the IT, muscle power performance was assessed through CMJ tests. In the 24-h before the two experimental sessions, athletes were asked to avoid any strenuous physical activity, consumption of caffeine, energy drinks, and alcohol.

### 2.2 Participants

*A priori* power analysis was performed using the G*Power software (v. 3.1.9.7; Heinrich Heine University in Düsseldorf, Germany) using the F test family (ANOVA: repeated measures, within factors), with four measurements. The analysis revealed that a total sample size of 11 participants would be sufficient to find medium effects of condition (effect size f = 0.25, α = 0.05) with an actual power of 0.8 (Ouergui et al., 2023a; Ouergui et al., 2023b). All athletes were recruited from the same club, to prevent potential interference/variation induced by training programs’ variation, based on the following inclusions criteria: 1) more than 5 years of experience in taekwondo; 2) be training at least five times a week; 3) not engage in any acute rapid weight loss strategies during the study period; 4) not having suffered muscle and joint injuries in the past 6 months; 5) not having taken drugs, medications or dietary supplements. Thirteen Italian taekwondo black belt athletes (7 males and 6 females; mean ± SD, age: 17.6 ± 2.7 years; 12.3 ± 2.2 years of practice; body height: 171.8 ± 4.8 cm; body mass: 59.1 ± 5.3 kg; body fat: 14.6% ± 8.2%) participated in this study. Athletes followed a standard training program of 8 weekly sessions (∼90 min/session) and were regularly engaged in national (i.e., national championships and cups) and international (i.e., G-1/E-1 and/or E-2 competitions, and/or European championships) competitions. In the week prior to the data collection, athletes conducted two familiarization sessions, with all the testing procedures, to minimize the learning effect (Chaabene et al., 2018), and the study was conducted during the competitive season. Athletes provided a written informed consent form after they were informed about the design of the study and the possible benefits and risks associated with it. For athletes under the age of 18, written informed consent was obtained from their parents or guardian. The study was approved by the Local Ethics Committee (University of Genoa, Italy. N. 2024/44) and was conducted according to the principles of the Declaration of Helsinki (World Medical Association, 2013).

### 2.3 Procedures

**FIGURE 1:**
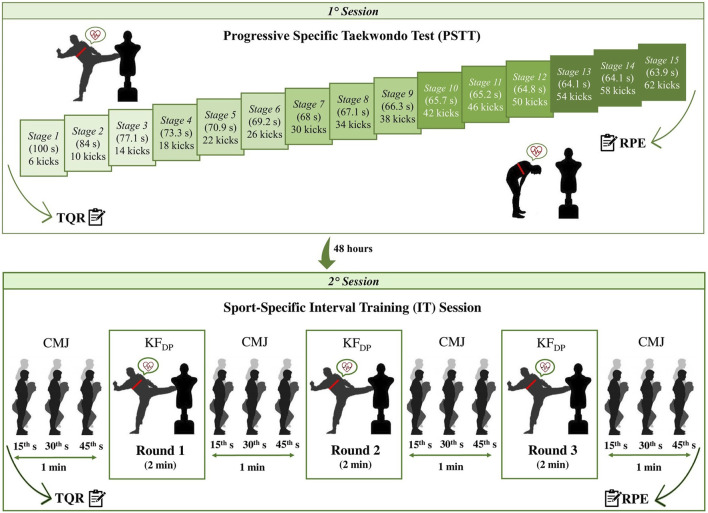
Schematic representation of the study design. Notes: TQR: total quality of recovery scale; RPE: rating of perceived exertion scale; s: second; CMJ: countermovement jump; KF_DP_: kick frequency at heart rate deflection point; min: minute.

Prior to the first session, body height and composition were measured with a stadiometer (*Seca Model 217; SECA GmbH & Co. KG., Hamburg, Germany*) and a bioelectrical impedance scale (*Tanita BC-420 MA; Tanita Corp., Tokyo, Japan*), with 0.1 cm and 0.1 kg resolution, respectively. Both experimental sessions were preceded by a standardized warm-up routine consisting of 2 min of stepping and moving according to the taekwondo-specific techniques and 3 min of low-intensity taekwondo kicks, for a total of 5 min (Mesquita et al., 2020; Albuquerque et al., 2022). After 5 min of passive recovery, athletes performed the PSTT and sport-specific IT (i.e., 3 rounds × 2 min/1 min of recovery in-between) at the KF_DP_, respectively. Before and after each round of the IT, three attempts of CMJ were allowed (Sant’Ana et al., 2021). Each attempt was made at the 15th, 30th, and 45th seconds of the recovery minute. Thus, a passive recovery interval of 15 s was applied between attempts. HR was continuously measured, beat-by-beat, during the PSTT and rounds of sport-specific IT. Total quality of recovery (TQR) (Kenttä and Hassmén, 1998) was assessed before, while rating of perceived exertion (RPE) (Borg, 1982) was evaluated after PSTT and sport-specific IT, respectively (Figure 1). The experimental sessions were conducted at the athletes’ sports center, by a researcher (a taekwondo coach, ≥20 years of taekwondo experience and black belt), at the same time of day (10:00–12:00 a.m.), under similar temperature and humidity conditions (25°C–27°C and 46%–47%, respectively) to avoid any influence of the circadian rhythms.

### 2.4 Measures

#### 2.4.1 Progressive specific taekwondo test

To perform the PSTT (Sant’Ana et al., 2019; Apollaro et al., 2024a), athlete executed the bandal-chagi (i.e., roundhouse kick) beginning with the right leg, in an area of about 2 × 2 m and using a punching bag equipped with a taekwondo body protector, positioned in same height of the athlete trunk. The first stage began with 6 kicks, alternating legs, and then progressively increasing 4 kicks on each new stage. During the test, athlete remained in step. The pace was dictated by sound signals with fixed intervals between each kick and with the intervals getting shorter every new stage, thus increasing the frequency of kicks. Sound signals were transmitted from a smartphone using the *ITStriker* app (*ETS4ME, São José, SC, Brazil*) containing the track of PSTT (Antonietto et al., 2021; Sant’Ana et al., 2021; Rodrigues et al., 2023; de Oliveira et al., 2024). Athlete was instructed to perform the bandal-chagi throughout the test, maintaining the same technical quality (it was considered adequate when the athlete lifted the knee, rotated the hips and delivered the kick horizontally into the target with the instep) and power of the kick (it was considered adequate when the instep impacted sonorously) as he/she would do in a competition to score points. Figure 1 details the duration and the frequency of kicks for every stage of the PSTT. The following criteria was used to establish end: 1) the athlete fails to track the KF (determined by beep and controlled by the researcher); 2) the athlete performs the kicks without maintaining the technical and/or power standard effectively (controlled by the researcher); or 3) the athlete stops the test (volitional exhaustion). A tolerance not exceeding 2 consecutive technical failures is allowed.

During the PSTT, the *ITStriker* app and a HR monitor strap (Polar H10; Polar Electro Oy, Kempele, Finland) paired with the app were used to record the following variables: 1) maximal heart rate (HR_MAX_) which is the heart rate recorded at the end of the test; 2) maximal kick frequency (KF_MAX_) which is the highest frequency of kicks reached in the last stage of the test; 3) heart rate deflection point (HR_DP_); 4) kick frequency at the HR_DP_ (KF_DP_); 5) total number of kicks (K_TOTAL_); 6) time to exhaustion.

**FIGURE 2:**
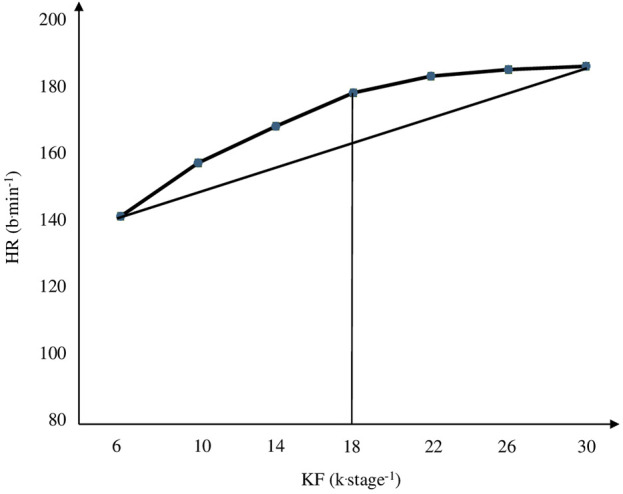
Graphical model of heart rate deflection point identification by D_MAX_ method (Kara et al., 1996). Notes: HR: heart rate; KF_DP_: kick frequency.

The HR_DP_, an indirect indicator of the anaerobic threshold (AT) (Bodner and Rhodes, 2000), was identified using the D_MAX_ method (Kara et al., 1996), similarly to previous studies (Sant’Ana et al., 2018; Sant’Ana et al., 2019; de Oliveira et al., 2024). According to de Assis Pereira et al. (2016), the D_MAX_ method does not present significant differences from maximal lactate steady state velocity and there was a high agreement between them (through Bland and Altman analysis). The HR curve was adjusted *versus* the KF obtained at each stage of the PSTT by a polynomial function of third order. Then, the first and last points of the curve were connected by a straight line, and the most distant point of the curve to the line was considered as the HR_DP_. Only values equal or greater than 140 b min^−1^ were used (Siahkouhian and Meamarbashi, 2013; de Assis Pereira et al., 2016). The KF at the stage corresponding to the HR_DP_ was called KF_DP_ (Figure 2).

#### 2.4.2 Sport-specific interval training session

Athlete performed a sport-specific IT (i.e., 3 rounds × 2 min/1 min of recovery in-between), at the KF_DP_ identified during the PSTT, using a punching bag equipped with a taekwondo body protector, positioned in same height of the athlete trunk. The *ITStriker* app was used in the “training mode” to ensure that the individual KF_DP_ of each athlete was maintained throughout all rounds (Sant’Ana et al., 2021). Thus, the pace was dictated by sound signals, transmitted from a smartphone using the app, with fixed intervals between each kick. During each round of IT, HR was measured, using the *ITStriker* app and a HR monitor strap (Polar H10; Polar Electro Oy, Kempele, Finland) paired with the app, to record round HR (HR_ROUND_) which is the HR recorded at the end of the round. To perform the IT, athlete executed the bandal-chagi (always alternating the legs and varying the technique with the front and back leg) and steps, hoopings, and movements characteristics of taekwondo between each kick. Athlete was instructed to perform the bandal-chagi throughout the IT, maintaining the same technical quality and power of the kick as he/she would do in a competition to score points.

#### 2.4.3 Countermovement jump test

Athlete performed the CMJ on an electronic contact mat (*Globus Ergo Jump; Globus Inc., Codognè, Italy*), with an accuracy of 0.01 m, to determine the maximum height of the vertical jump. Each athlete was instructed to stand on mat while keeping their hands on their hips. After that, he/she was asked to rapidly perform a downward squat movement and to jump vertically for maximum height after a verbal command. Before and after each round of the sport-specific IT, three attempts of CMJ were performed, and the highest jump height of each period was used in the data analysis (Sant’Ana et al., 2021). Each attempt was made at the 15th, 30th, and 45th seconds of the recovery minute. Thus, a passive recovery interval of 15 s was applied between attempts. The researcher provided standard verbal encouragement to all athletes during the CMJ tests. Within-session reliability of CMJ measures was computed using the intraclass correlation coefficient (ICC = 0.997, 95% CI = 0.993–0.999, *p* < 0.001) and the coefficient of variation (CV = 4.5%). Flight time was measured using the contact mat and jump height (cm) was calculated as follows: 9.81 × flight time^2^/8 (Bosco et al., 1983). CMJ jump height and the athletes’s body mass were used to calculate absolute peak power by the following equation of Sayers et al. (1999): absolute peak power (W) = 60.7 × jump height (cm) + 45.3 × body mass (kg) – 2055.

#### 2.4.4 Perceptual measures

Recovery state was recorded 15 min before each experimental session using the TQR scale (a.u.) (Kenttä and Hassmén, 1998), which ranged from “very very poor recovery” (value 6) to “very very good recovery” (value 20). Immediately at the end (Tayech et al., 2020; Tayech et al., 2022) of PSTT and sport-specific IT, perceived exertion was evaluated using the Borg 6–20 RPE scale (a.u.) (Borg, 1982), which ranged from “very, very light” (value 6) to “*very, very hard*” (value 20).

### 2.5 Statistical analysis

Data analyses were performed using *Jamovi* software (*v. 2.3.28*; The Jamovi Project, Australia). Within-session reliability of CMJ test measures was computed using an average measures two-way random ICC with absolute agreement and 95% confidence intervals, and the CV. The ICC values were interpreted as follows: <0.5, poor; 0.5–0.75, moderate; 0.75–0.9, good; >0.9, excellent (Koo and Li, 2016). While, CV values were interpreted as follows: <5%: excellent agreement; <10%: very good agreement; <15%: acceptable; >15%: poor (Hopkins et al., 2009). The Shapiro–Wilk test revealed the normal distribution of all the considered variables. Therefore, data are presented as mean ± standard deviation [95% confidence intervals]. Pearson’s correlation coefficient (*r*), with 95% confidence intervals, was used to examine relationships between HR_DP_, identified during the PSTT, and HR responses during the rounds of sport-specific IT at the KF_DP_. The magnitude of correlations was assessed using the following benchmarks: <0.1, trivial; 0.1–0.3, low; 0.3–0.5, moderate; 0.5–0.7, large; 0.7–0.9, very large; >0.9, nearly perfect; = 1, perfect (Hopkins et al., 2009). Repeated-measures ANOVA were performed, with Bonferroni’s *post hoc* tests, to compare the HR_DP_, identified during the PSTT, with HR responses, as well as the CMJ performance, during the rounds of sport-specific IT at the KF_DP_. Assumption of sphericity was assessed using Mauchly test. A Greenhouse-Geisser correction factor was applied to the error and factor degrees of freedom if sphericity was violated. Partial eta-squared (η^2^_p_) was used as a measure of effect size for the repeated-measures ANOVA, and values were interpreted as: <0.06, small; 0.06–0.14, moderate; >0.14, large (Cohen, 1988). In addition, Cohen’s *d* was used as an effect size measure for the paired data and were graded as: <0.20, trivial; 0.20–0.59, small; 0.60–1.19, moderate; 1.20–2.00, large; >2.0, very large (Hopkins et al., 2009). Mean changes in CMJ performance after each round of the IT were compared with the smallest worthwhile change (SWC) calculated using baseline CMJ data as: 0.2 × between-subject SD; and minimal detectable change (MDC_95%_) determined as: standard error of measurement (SEM) × 1.96 × √2 (Weir, 2005; Haley and Fragala-Pinkham, 2006; Hopkins et al., 2009). Lasty, Pearson’s correlation coefficient (*r*), with 95% confidence intervals, was also used to investigate relationships of perceptual measures (TQR and RPE) with HR responses and CMJ performance during the rounds of sport-specific IT. The magnitude of correlations was assessed as previously indicated. The statistical significance was accepted when *p* < 0.05.

## 3 Results

Table 1 shows PSTT performance in the taekwondo athletes tested.

**TABLE 1:** Descriptive statistics of Progressive Specific Taekwondo Test performance in taekwondo athletes (values are presented as mean ± standard deviation [95% confidence interval], n = 13).

Variable	Mean ± SD [95% CI]
Progressive specific taekwondo test	
TQR (a.u.)	18 ± 2 [17–19]
HR_MAX_ (b^.^min^-1^)	191 ± 5 [188–194]
KF_MAX_ (k^.^stage^−1^)	47 ± 6 [43–50]
KF_MAX_ (k^.^min^-1^)	43 ± 5 [40–47]
HR_DP_ (b^.^min^-1^)	169 ± 9 [163–175]
HR_DP_ (%HR_MAX_)	88 ± 4 [86–91]
KF_DP_ (k^.^stage^−1^)	23 ± 4 [21–25]
KF_DP_ (k^.^min^-1^)	20 ± 4 [18–22]
KF_DP_ (%KF_MAX_)	46 ± 7 [42–50]
K_TOTAL_ (n°)	303 ± 68 [262–344]
Time to exhaustion (s)	820 ± 94 [763–876]
RPE (a.u.)	18 ± 2 [17–19]

Notes: TQR: total quality of recovery scale; HR_MAX_: maximal heart rate; KF_MAX_: maximal kick frequency; HR_DP_: heart rate deflection point; KF_DP_: kick frequency at heart rate deflection point; K_TOTAL_: total number of kicks; RPE: rating of perceived exertion scale.

Before the second experimental session, TQR recorded values of 17 ± 2 [16–18] a.u. During the entire sport-specific IT, athletes performed 116 ± 22 [103–129] kicks. RPE values, collected immediately at the end of the sport-specific IT, were 10 ± 2 [9–12] a.u.

**FIGURE 3:**
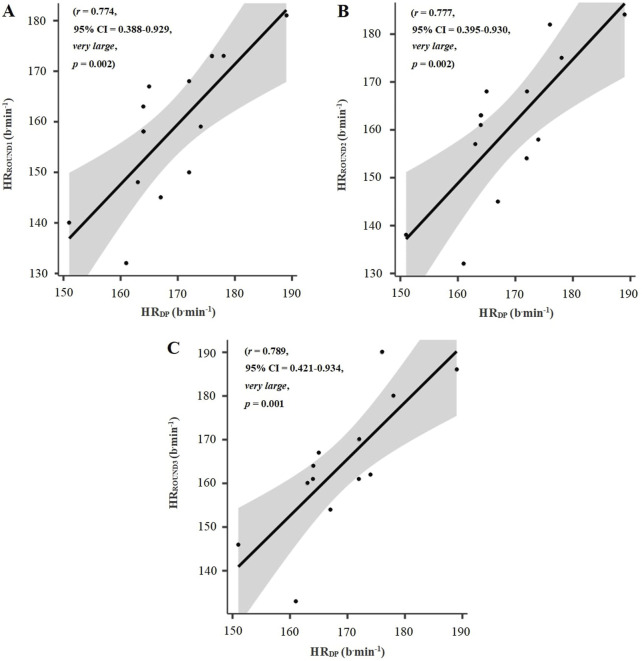
Scatterplot diagrams for the relationship between heart rate deflection point, identified during the Progressive Specific Taekwondo Test, and heart rate responses during the rounds 1 **(A)**, 2 **(B)**, and 3 **(C)** of sport-specific interval training at the kick frequency at heart rate deflection point in taekwondo athletes (n = 13). Notes: HR_DP_: heart rate deflection point; HR_ROUND1_: HR during round 1; HR_ROUND2_: HR during round 2; HR_ROUND3_: HR during round 3.

Figure 3 reports the correlations between HR_DP_, identified during the PSTT, and HR responses during the three rounds of sport-specific IT at the KF_DP_.

Table 2 presents the HR responses during the three rounds of sport-specific IT at the KF_DP_, identified during the PSTT. A main effect was found for HR responses during the rounds of sport-specific IT (F_9,941_ = 1.487; *p* = 0.003; η^2^_p_ = 0.453, *large*). HR was significantly lower in round 1 (*p* = 0.008; *d* = 1.14, *moderate*) than HR_DP_. In contrast, no difference was observed between the HR of rounds 2 (*p* = 0.067; *d* = 0.83, *moderate*) and 3 (*p* = 0.653; *d* = 0.48, *small*) and HR_DP_. No difference emerged between the HR of round 1 and 2 (*p =* 0.352; *d* = 0.58, *small*). In contrast, HR was significantly lower in rounds 1 (*p* = 0.011; *d* = 1.11, *moderate*) and 2 (*p* = 0.009; *d* = 1.13, moderate) than in round 3.

**TABLE 2:** Descriptive statistics of heart rate responses during the three rounds of sport-specific interval training at the kick frequency at heart rate deflection point, identified during the Progressive Specific Taekwondo Test (values are presented as mean ± standard deviation [95% confidence interval], n = 13).

	PSTT	Sport-specific interval training		
	HR_DP_	HR_ROUND1_	HR_ROUND2_	HR_ROUND3_
HR (b^.^min^-1^)	169 ± 9 [163–175] †	158 ± 14 [149–167]	160 ± 16 [151–170]	164 ± 16 [155–174]‡

Notes: PSTT: progressive specific taekwondo test; HR: heart rate; HR_DP_: heart rate deflection point; HR_ROUND1_: HR, at the end of round 1; HR_ROUND2_: HR, at the end of round 2; HR_ROUND3_: HR, at the end of round 3. †: Significantly different from HR_ROUND1_ (*p* < 0.01); ‡: Significantly different from HR_ROUND1_ and HR_ROUND2_ (*p* < 0.01).

Table 3 shows the CMJ performance pre, post, and between the interval of the rounds of sport-specific IT at the KF_DP_, identified during the PSTT. A main effect was found for CMJ performance during sport-specific IT (F_4,144_ = 3; *p* = 0.013; η^2^_p_ = 0.257, *large*). No difference was observed between pre- and post-IT CMJ performance (*p* = 0.210; *d* = 0.66, *moderate*), as well as between pre- and interval CMJ performance of rounds 1 (*p* = 1.000; *d* = 0.06, *trivial*) and 2 (*p* = 1.000; *d* = 0.21, *small*). No difference emerged between CMJ performance in round 1 interval and that in round 2 (*p* = 1.000; *d* = 0.33, *small*), while CMJ performance in round 1 interval was significantly lower (*p* = 0.009; *d* = 1.13, *moderate*) than that post-IT. In contrast, CMJ performance in the round 2 interval showed no difference with that post-IT (*p* = 0.236; *d* = 0.64, *moderate*).

**TABLE 3:** Descriptive statistics of CMJ performance pre, post, and between the interval of the rounds of sport-specific interval training at the kick frequency at heart rate deflection point, identified during the Progressive Specific Taekwondo Test (values are presented as mean ± standard deviation [95% confidence interval], n = 13).

	Sport-specific interval training			
	CMJ_PRE_	CMJ_REST1_	CMJ_REST2_	CMJ_POST_
CMJ (W)	2602 ± 528 [2284–2921]	2596 ± 547 [2266–2927]	2620 ± 561 [2281–2959]	2672 ± 537 [2347–2996] †

Notes: CMJ: countermovement jump; CMJ_PRE_: CMJ, performance pre-IT; CMJ_REST1_: CMJ, performance during interval between rounds 1 and 2; CMJ_REST2_: CMJ, performance during interval between rounds 2 and 3; CMJ_POST_: CMJ, performance post-IT., Significantly different from CMJ_REST1_ (*p* < 0.01).

SWC_0.2_, SEM, and MDC_95%_ for baseline CMJ performance were 106 W, 55 W, and 153 W, respectively. The mean changes in CMJ performance, compared with baseline, were the following: Δ CMJ_REST1_–CMJ_PRE_ = −6 W, −0.3%; Δ CMJ_REST2_–CMJ_PRE_ = 18 W, 0.5%; Δ CMJ_POST_–CMJ_PRE_ = 69 W, 2.7%.

Table 4 reports the correlations of perceptual measures (TQR and RPE) with HR responses and CMJ performance during the rounds of sport-specific IT at the KF_DP_.

**TABLE 4:** Correlation coefficients of perceptual measures with heart rate responses during the three rounds and CMJ performance pre, post, and between the interval of the rounds of sport-specific interval training at the kick frequency at heart rate deflection point in taekwondo athletes (n = 13).

	CMJ_PRE_ (W)	HR_ROUND1_ (b^.^min^-1^)	CMJ_REST1_ (W)	HR_ROUND2_ (b^.^min^-1^)	CMJ_REST2_ (W)	HR_ROUND3_ (b^.^min^-1^)	CMJ_POST_ (W)	RPE (a.u.)
TQR (a.u.) *r* [95% CI] *Magnitude p*-value	0.011 [-0.543–0.559] *trivial* 0.971	0.241 [-0.358–0.699] low 0.428	−0.057 [-0.590–0.510] *trivial* 0.853	0.207 [-0.389–0.680] low 0.498	−0.016 [-0.562–0.540] *trivial* 0.958	0.166 [-0.423–0.657] low 0.587	−0.050 [-0.585–0.515] *trivial* 0.871	−0.365 [-0.763–0.233] *moderate* 0.220
RPE (a.u.) *r* [95% CI] *Magnitude p*-value	−0.301 [-0.731–0.300] *moderate* 0.318	0.519 [-0.044–0.832] *large* 0.069	−0.313 [-0.737–0.288] *moderate* 0.298	0.573 [0.032–0.854] *large* 0.041	−0.304 [-0.732–0.297] *moderate* 0.313	0.642 [0.141–0.881] *large* 0.018	−0.305 [-0.733–0.296] *moderate* 0.311	–

Notes: TQR: total quality of recovery scale; RPE: rating of perceived exertion scale; CMJ: countermovement jump; CMJ_PRE_: CMJ, performance pre-IT; HR: heart rate; HR_ROUND1_: HR, at the end of round 1; CMJ_REST1_: CMJ, performance during interval between rounds 1 and 2; HR_ROUND2_: HR, at the end of round 2; CMJ_REST2_: CMJ, performance during interval between rounds 2 and 3; HR_ROUND3_: HR, at the end of round 3. CMJ_POST_: CMJ, performance post-IT.

## 4 Discussion

The aim of this study was to prescribe a sport-specific IT session (which mimics the time structure of the official match), at the KF_DP_ identified during the PSTT, using the technical action of bandal-chagi, and to investigate HR response and muscle power performance, through CMJ tests, in taekwondo athletes. The first hypothesis was confirmed as the rounds 2 and 3 of sport-specific IT at KF_DP_ elicited HR responses similar to those observed at KF_DP_ during the PSTT as the required intensity corresponded to ∼88% of the HR_MAX_. In this context, the HR was significantly lower in IT round 1 than the HR_DP_, due to the slow component of cardiovascular kinetics (i.e., to HR evolution as a function of time and exercise intensity) typical of exercise performed in the heavy domain (Jamnick et al., 2020). On the other hand, HR_DP_ was significantly and positively correlated with HR responses recorded during each round of the IT. The second hypothesis was also confirmed as the three rounds of sport-specific IT at KF_DP_ did not cause a decrease in muscle power performance assessed through CMJ tests. Specifically, no difference was observed between pre- and post-IT CMJ performance, as well as between pre- and interval CMJ performance of rounds 1 and 2, supporting the idea that the KF_DP_ does not impair the neuromuscular system, in the high force production in small time intervals and in the recruitment of fast twitch muscle fibers, during the repeated bandal-chagi technical action (Sant’Ana et al., 2021).

Similarly to the aim of our study, Sant’Ana et al. (2021) investigated the acute effects of a sport-specific IT session (which mimics the time structure of the official match), performed at the KF_DP_ found in the PSTT, on HR responses and CMJ test performance. They found that only the HR in IT round 1 was significantly lower at HR_DP_. In contrast, rounds 2 and 3 elicited HR responses similar to those observed at KF_DP_ during the PSTT and significantly greater than that of round 1 (Sant’Ana et al., 2021). In this context, although Sant’Ana et al. (2021) also documented expected HR responses, it is important to highlight that they allowed athletes during the IT to perform kicks with technical variations and combining up to three kicks per sound signal that might have altered the KF required of the athlete by generating intensity-specific responses. In contrast, in our study athletes performed the single technical action of bandal-chagi for each sound signal to reproduce the same KF required to athletes at HR_DP_ during the PSTT, with deep objective to provide a basic methodological control step and consequently incentivize the use of HR_DP_ and KF_DP_ to tailor the intensity of IT. Therefore, the current results support in the first instance the validity of the aerobic capacity indicators derived in the PSTT, to prescribe sport-specific training, and provide a basis for understanding and structuring future ITs that reflect more closely the technical-tactical demands of competitions.

In line with our results, Sant’Ana et al. (2021) found that the three rounds of sport-specific IT at KF_DP_ did not cause a decrease in muscle power performance as no difference was observed between pre- and post-IT CMJ performance, as well as between pre- and interval CMJ performance of rounds 1 and 2. In this context, if kicks with technical variations and up to three kicks for each sound signal, in the IT protocol implemented by Sant’Ana et al. (2021), did not cause a decrease in muscle power performance, it was consequently conceivable that the single technical action of bandal-chagi for each sound signal would provide similar results. Recent studies have documented the positive influence of muscle power, as assessed by the CMJ test, on the ability to repeat high-intensity intermittent efforts in an anaerobic sport-specific taekwondo test (Albuquerque et al., 2022; Apollaro et al., 2024c). In addition, Sant’Ana et al. (2017) found a significant decrease in bandal-chagi impact after a sport-specific time-to-exhaustion test performed at the KF_MAX_ found in the PSTT. In this sense, the fact the KF_DP_ does not impair muscle power performance and does not generate fatigue is fundamental to support the validity of HR_DP_ and KF_DP_, derived from PSTT, for the prescription of sport-specific IT directed at aerobic capacity conditioning in taekwondo. Additionally, Sant’Ana et al. (2021) also found that after the three rounds of the IT there was a 3.1% improvement in CMJ performance compared with pre, by assuming a post-activation potentiation effect. Similarly, the present study showed that after three rounds of the IT there was a 2.7% improvement in CMJ performance compared with pre. However, the comparison of this increase with SWC_0.2_ and MDC_95%_ did not show a useful change such as to assume a practical change related to a post-activation potentiation effect.

In addition, we also investigated the relationships between the perceptual measures, recorded pre- and post-IT (i.e., TQR and RPE, respectively), and heart rate responses during the three rounds and CMJ performance pre, post, and between the interval between rounds. No significant relationship emerged between TQR and heart rate responses, as well as between TQR and CMJ performance. The TQR reflects the athlete’s general and subjective psycho-physical state and may therefore not be a good predictor of immediate physiological response. In fact, HR can be influenced by several factors other than exercise intensity (e.g., warm-up, environmental temperature, hydration, etc.), as well as the current state of the neuromuscular system may reflect the type of activity performed and the level of motivation. In parallel, significant relationships emerged between RPE and HR recorded during rounds 2 and 3 of the IT, while no significant relationships emerged between RPE and CMJ performance. In this context, it is important to highlight that the relationship between RPE and HR tends to increase in magnitude round by round. This trend suggests a possible alignment between physiological and perceived exertion load with the intensification of cardiovascular demands throughout the rounds. On the other hand, an inverse relationship logically characterizes the relationship between RPE and CMJ performance, although the lack of significance supports the idea that KF_DP_ does not compromise the neuromuscular system. Finally, no significant relationship emerged between TQR and RPE. This could be justified by the fact that the TQR may be underestimated or overestimated by athletes as it is recorded before training and therefore relates to a relatively long and general period of time. On the contrary, RPE is recorded immediately after a specific type of exercise, thus reflecting the situation at that moment.

The typical intermittency of combat activity generates post-match official [La] values ranging from 6.7 ± 2.5 to 14.0 ± 4.2 mmol L^−1^ (Apollaro et al., 2024b), and during the day of competition the athlete could perform up to 4–6 times. Therefore, the oxidative system is attributed a central role in supporting the recovery process between matches, particularly in the recovery of pH (Apollaro et al., 2024a). In this sense, the prescription of sport-specific training directed at improving aerobic capacity in the taekwondo athletes is one important aspect for performance improvement in this sport and should be based on carefully determined thresholds and training zones (Sant’Ana et al., 2019; Sant’Ana et al., 2021). In this context, the next step of research in this field should focus on studying long-term adaptations to this type of training. Sant’Ana et al. (2011) tested the effects of sport-specific IT (i.e., 4 rounds × 3 min/3 min of active recovery in-between), at a stage higher than KF_DP_ and combined with strength and technical-tactical training, on PSTT aerobic capacity and power indicators after an 8-week training period. They found no changes in aerobic capacity indicators (but a strong trend toward significance for KF_DP_), while they did find a significant increase in KF_MAX_ and K_TOTAL_, indicating an improvement in aerobic power. Although this study is the only evidence currently available and involved a very limited number of athletes, it is reasonable to assume that sport-specific aerobic training can improve key aerobic indicators. On the other hand, it is important to highlight that given the substantial increase in the number of national and international level competitions, sport-specific aerobic capacity training can be a valuable tool for coaches in the post-competition period to promote optimal recovery (Apollaro et al., 2024a). Moreover, during the off-season, when general conditioning methods such as running are usually emphasized, this type of training can be a more specific alternative, allowing athletes to maintain technical engagement, improve aerobic efficiency, and elicit specific energy dynamics (Sant’Ana et al., 2011; dos Santos et al., 2018). Among the sport-specific tests currently available for the assessment of endurance in taekwondo, the PSTT is the most studied and used in practice (Apollaro et al., 2024a). It allows the determination of both aerobic capacity (i.e., HR_DP_ and KF_DP_) and power indicators (i.e., HR_MAX_ and KF_MAX_), based on HR, useful for the prescription of sport-specific training (Sant’Ana et al., 2017; Sant’Ana et al., 2021). In the present study, the HR_DP_ was identified at ∼88% of the HR_MAX_, in line with previously reported in taekwondo athletes during the execution of the PSTT (Sant’Ana et al., 2009; Sant’Ana et al., 2011; dos Santos et al., 2018; Sant’Ana et al., 2018) and within the range documented in the literature (Ribeiro et al., 1985; Bunc et al., 1995; Bodner and Rhodes, 2000).

### 4.1 Limitations and future directions

First, the sample, although not underpowered, includes a limited number of national/international athletes from the junior/senior age categories, which limits the generalizability of the results to athletes from other competitive level (i.e., state/regional) and/or age categories (i.e., cadets). Second, we prescribed a sport-specific IT, which mimics the time structure of the official match, only at the KF_DP_. In this regard, it might be appropriate to investigate HR responses and muscle power performance also at KF immediately above and below KF_DP_, to further support the practical validity of aerobic capacity indicators and the results of our study. In parallel, sport-specific ITs with a longer time structure than the official match should also be prescribed, as the duration of the sessions is an aspect to be considered for the improvement of aerobic capacity and the interpretation of HR responses and muscle power performance. Third, it was previously found that the HR_DP_ and KF_DP_ in the PSTT were not different and significantly correlated with the HR_AT_ and KF_AT_ during a sport-specific constant load test at the fixed concentration of 4 mmol^.^L^−1^ of [La] (Sant’Ana et al., 2009). However, measurements of [La] and VO_2_ should be included to provide a direct comparative analysis to improve the experimental external validity and to expand the understanding of internal load with the aim of supporting the validity of HR_DP_ as an indirect indicator of AT. Finally, Sant’Ana et al. (2017) documented test-retest ICCs of 0.98 and 0.97 for the KF_MAX_ and HR_MAX_, respectively, in the PSTT. However, the implementation of electronic body protector and the elimination of the skipping between kicks could objectify the high- and low-intensity phases to the benefit of test-retest reliability (Apollaro et al., 2024a). Consequently, it would also become possible to study inter-/intra-rater reliability of the PSTT, since the evaluator’s task would be limited to just checking the KF.

### 4.2 Practical applications

The HR_DP_ and KF_DP_, identified during the PSTT, can be used as valid indicators of aerobic capacity to prescribe a sport-specific IT that mimics the time structure of the official match. In this regard, the current and previous findings (Sant’Ana et al., 2021) suggest that the technical variation and the number of kicks for each sound signal need an appropriate adjustment of the KF, based on the estimated time to perform the different techniques (Falcó et al., 2011), to ensure that the attack/skipping ratio of the KF_DP_ is properly maintained by consequently generating distinct physiological responses. Finally, the *ITStriker* app is a low-cost tool that can be easily used during the execution of the PSTT to automatically derive internal and external load parameters and for their subsequent use in “training mode”.

## 5 Conclusion

The PSTT allows the determination of both aerobic capacity (i.e., HR_DP_ and KF_DP_) and power indicators (i.e., HR_MAX_ and KF_MAX_) useful for subsequent prescription of sport-specific training. In the present study, we prescribed an IT session (which mimics the time structure of the official match), at the KF_DP_ identified during the PSTT, in which athletes performed the single technical action of bandal-chagi for each sound signal. During IT rounds, expected HR responses emerged and muscle power performance was not compromised. Overall, the present results support the validity of aerobic capacity indicators, derived from the PSTT and based on heart rate, for the prescription of sport-specific training in taekwondo.

## Data Availability

The raw data supporting the conclusions of this article will be made available by the authors, without undue reservation.

## References

[B1] Albuquerque M. R. Tavares L. D. Longo A. R. Caldeira Mesquita P. H. Franchini E. (2022). Relationship between indirect measures of aerobic and muscle power with frequency speed of kick test multiple performance in taekwondo athletes. Int. J. Sports Med. 43 (3), 254–261. 10.1055/a-1546-9221 34388844

[B2] Antonietto N. R. dos Santos D. A. Costa K. F. Fernandes J. R. Carrenho Queiroz A. C. Valenzuela Perez D. I. (2021). Beetroot extract improves specific performance and oxygen uptake in taekwondo athletes: a double-blind crossover study. Ido Mov. Cult. 21 (4), 12–19. 10.14589/ido.21.4.3

[B3] Apollaro G. Franchini E. Falcó C. Detanico D. Kons R. L. (2024a). Sport-specific tests for endurance in taekwondo: a narrative review with guidelines for the assessment. Strength Cond. J. 46 (6), 627–645. 10.1519/SSC.0000000000000828 PMC11511156 39453244

[B4] Apollaro G. Ouergui I. Rodríguez Y. Q. Kons R. L. Detanico D. Franchini E. (2024b). Anaerobic sport-specific tests for taekwondo: a narrative review with guidelines for the assessment. Sports 12 (10), 278. 10.3390/sports12100278 39453244 PMC11511156

[B5] Apollaro G. Panascì M. Ouergui I. Franchini E. Falcó C. Ruggeri P. (2024c). Influence of body composition and muscle power performance on multiple frequency speed of kick test in taekwondo athletes. Sports 12 (12), 322. 10.3390/sports12120322 39728862 PMC11679010

[B6] Araujo M. P. Soares P. P. Hausen M. R. Julio H. S. Porto F. Gurgel J. L. (2021). Validity of an interval taekwondo-specific cardiopulmonary exercise test. J. Strength Cond. Res. 35 (7), 1956–1963. 10.1519/JSC.0000000000002988 30676393

[B7] Bodner M. E. Rhodes E. C. (2000). A review of the concept of the heartrate deflection point. Sports Med. 30 (1), 31–46. 10.2165/00007256-200030010-00004 10907756

[B8] Borg G. A. (1982). Psychophysical bases of perceived exertion. Med. Sci. Sports Exerc. 14 (5), 377–381. 10.1249/00005768-198205000-00012 7154893

[B9] Bosco C. Luhtanen P. Komi P. V. (1983). A simple method for measurement of mechanical power in jumping. Eur. J. Appl. Physiol. Occup. Physiol. 50 (2), 273–282. 10.1007/BF00422166 6681758

[B10] Bridge C. A. Ferreira da Silva Santos J. Chaabène H. Pieter W. Franchini E. (2014). Physical and physiological profiles of taekwondo athletes. Sports Med. 44 (6), 713–733. 10.1007/s40279-014-0159-9 24549477

[B11] Bunc V. Hofmann P. Leitner H. Gaisl G. (1995). Verification of the heart rate threshold. Eur. J. Appl. Physiol. 70 (3), 263–269. 10.1007/BF00238574 7607203

[B12] Chaabene H. Negra Y. Bouguezzi R. Capranica L. Franchini E. Prieske O. (2018). Tests for the assessment of sport-specific performance in olympic combat sports: a systematic review with practical recommendations. Front. Physiol. 9 (10), 386. 10.3389/fphys.2018.00386 29692739 PMC5902544

[B13] Cohen J. (1988). Statistical power anaylsis for the behavioural science. 2nd ed. Hillsdale, NJ, USA: Lawrence Earlbaum Associates.

[B14] Conconi F. Grazzi G. Casoni I. Guglielmini C. Borsetto C. Ballarin E. (1996). The conconi test: methodology after 12 years of application. Int. J. Sports Med. 17 (7), 509–519. 10.1055/s-2007-972887 8912066

[B15] de Assis Pereira P. E. Piubelli Carrara V. K. Mello R. G. Pereira D. J. M. Fernandes Guerra R. L. Silva Marques de Azevedo P. H. (2016). The relationship between the heart rate deflection point test and maximal lactate steady state. J. Sports Med. Phys. Fit. 56 (5), 497–502. 26014090

[B16] de Oliveira L. B. Sant’ Ana J. Freccia G. W. Coswig V. S. Diefenthaeler F. (2024). Validity of a mobile-based specific test to estimate metabolic thresholds in boxers. Proc. Inst. Mech. Eng. P. 238 (1), 15–22. 10.1177/17543371221084563

[B17] dos Santos F. F. Sant’ Ana J. de Carvalho R. S. da Silva E. C. da Costa E. L. (2018). Avaliacao da capacidade e potencia aerobia de atletas de taekwondo do amazonas em teste especifico. Rev. Bras. Prescr. Fisiol. Exerc. 12 (72), 89–95. Available online at: https://www.rbpfex.com.br/index.php/rbpfex/article/view/1348.

[B18] Falcó C. Estevan I. Vieten M. (2011). Kinematical analysis of five different kicks in taekwondo. Port. J. Sport Sci. 11 (2), 219–222.

[B19] Haley S. M. Fragala-Pinkham M. A. (2006). Interpreting change scores of tests and measures used in physical therapy. Phys. Ther. 86 (5), 735–743. 10.1093/ptj/86.5.735 16649896

[B20] Hausen M. Soares P. P. Araujo M. P. Esteves D. Julio H. Tauil R. (2018). Eliciting higher maximal and submaximal cardiorespiratory responses during a new taekwondo-specific aerobic test. Int. J. Sports Physiol. Perform. 13 (10), 1357–1364. 10.1123/ijspp.2017-0846 29745772

[B21] Hopkins W. G. Marshall S. W. Batterham A. M. Hanin J. (2009). Progressive statistics for studies in sports medicine and exercise science. Med. Sci. Sports Exerc. 41 (1), 3–13. 10.1249/MSS.0b013e31818cb278 19092709

[B22] Jamnick N. A. Pettitt R. W. Granata C. Pyne D. B. Bishop D. J. (2020). An examination and critique of current methods to determine exercise intensity. Sports Med. Auckl. N.Z. 50 (10), 1729–1756. 10.1007/s40279-020-01322-8 32729096

[B23] Kara M. Gökbel H. Bedìz C. Ergene N. Uçok K. Uysal H. (1996). Determination of the heart rate deflection point by the dmax method. J. Sports Med. Phys. Fit. 36 (1), 31–34. 8699835

[B24] Kenttä G. Hassmén P. (1998). Overtraining and recovery. A conceptual model. Sports Med. 26 (1), 1–16. 10.2165/00007256-199826010-00001 9739537

[B25] Koo T. Li M. (2016). A guideline of selecting and reporting intraclass correlation coefficients for reliability research. J. Chiropr. Med. 15 (2), 155–163. 10.1016/j.jcm.2016.02.012 27330520 PMC4913118

[B26] Mesquita P. H. C. Franchini E. Romano-Silva M. A. Lage G. M. Albuquerque M. R. (2020). Transcranial direct current stimulation: no effect on aerobic performance, heart rate, or rating of perceived exertion in a progressive taekwondo-specific test. Int. J. Sports Physiol. Perform. 15 (7), 958–963. 10.1123/ijspp.2019-0410 32023547

[B27] Ouergui I. Delleli S. Messaoudi H. Bridge C. A. Chtourou H. Franchini E. (2023a). Effects of conditioning activity mode, rest interval and effort to pause ratio on post-activation performance enhancement in taekwondo: a randomized study. Front. Physiol. 14 (12), 1179309. 10.3389/fphys.2023.1179309 37501925 PMC10369352

[B28] Ouergui I. Franchini E. Messaoudi H. Chtourou H. Bouassida A. Bouhlel E. (2021). Effects of adding small combat games to regular taekwondo training on physiological and performance outcomes in Male young athletes. Front. Physiol. 12 (1), 646666. 10.3389/fphys.2021.646666 33868014 PMC8047306

[B29] Ouergui I. Jebabli A. Messaoudi H. Delleli S. Chtourou H. Bouassida A. (2023b). The effects of tempo and loudness variations during warm-up with music on perceived exertion, physical enjoyment and specific performances in Male and female taekwondo athletes. PLoS One 18 (4), e0284720. 10.1371/journal.pone.0284720 37104494 PMC10138780

[B30] Ouergui I. Messaoudi H. Chtourou H. Wagner M. O. Bouassida A. Bouhlel E. (2020). Repeated sprint training vs. repeated high-intensity technique training in adolescent taekwondo athletes - a randomized controlled trial. Int. J. Environ. Res. Public Health 17 (12), 4506. 10.3390/ijerph17124506 32585907 PMC7345419

[B31] Ribeiro J. P. Fielding R. A. Hughes V. Black A. Bochese M. A. Knuttgen H. G. (1985). Heart rate break point May coincide with the anaerobic and not the aerobic threshold. Int. J. Sports Med. 6 (4), 220–224. 10.1055/s-2008-1025844 4044107

[B32] Rocha F. P. Louro H. Matias R. Brito J. Costa A. M. (2016). Determination of aerobic power through a specific test for taekwondo - a predictive equation model. J. Hum. Kinet. 53 (14), 117–126. 10.1515/hukin-2016-0016 28149417 PMC5260582

[B33] Rodrigues J. C. C. Penna E. M. Machado H. E. S. Sant’ Ana J. Diefenthaeler F. Coswig V. S. (2023). Effects of lower and upper body fatigue in striking response time of amateur karate athletes. PeerJ 11 (31), e14764. 10.7717/peerj.14764 36743962 PMC9897062

[B34] Sant’Ana J. da Silva J. F. Guglielmo L. G. A. (2009). Variáveis fisiológicas identificadas em teste progressivo específico para taekwondo. Mot. Rev. Educ. Fis. 15 (3), 611–620. 10.5016/2113

[B35] Sant’Ana J. Franchini E. da Silva V. Diefenthaeler F. (2017). Effect of fatigue on reaction time, response time, performance time, and kick impact in taekwondo roundhouse kick. Sports Biomech. 16 (2), 201–209. 10.1080/14763141.2016.1217347 27592682

[B36] SantʼAna J. Franchini E. Murias J. M. Diefenthaeler F. (2019). Validity of a taekwondo-specific test to measure VO2peakand the heart rate deflection point. J. Strength Cond. Res. 33 (9), 2523–2529. 10.1519/JSC.0000000000002153 28737589

[B37] Sant’Ana J. Franchini E. Sakugawa R. L. Diefenthaeler F. (2018). Estimation equation of maximum oxygen uptake in taekwondo specific test. Sport Sci. Health 14, 699–703. 10.1007/s11332-018-0502-x

[B38] Sant’Ana J. Liberali R. Navarro F. (2011). Treinamento de resistencia aerobia para atletas de taekwondo. Rev. Bras. Prescr. Fisiol. Exerc 5 (28), 308–316. Available online at: https://www.rbpfex.com.br/index.php/rbpfex/article/view/346.

[B39] Sant’Ana J. Sakugawa R. L. Diefenthaeler F. (2021). The effect of a pace training session on internal load and neuromuscular parameters in taekwondo athletes. Front. Physiol. 12 (3), 710627. 10.3389/fphys.2021.710627 34413790 PMC8370830

[B40] Sayers S. P. Harackiewicz D. V. Harman E. A. Frykman P. N. Rosenstein M. T. (1999). Cross-validation of three jump power equations. Med. Sci. Sports Exer. 31 (4), 572–577. 10.1097/00005768-199904000-00013 10211854

[B41] Siahkouhian M. Meamarbashi A. (2013). Advanced methodological approach in determination of the heart rate deflection point: s.dmax *versus* l.Dmax methods. J. Sports Med. Phys. Fit. 53 (1), 27–33. 23470908

[B42] Taati B. Arazi H. Bridge C. A. Franchini E. (2022). A new taekwondo-specific field test for estimating aerobic power, anaerobic fitness, and agility performance. PLoS One 17 (3), e0264910. 10.1371/journal.pone.0264910 35294451 PMC8926267

[B43] Tayech A. Mejri M. A. Chaouachi M. Chaabene H. Hambli M. Brughelli M. (2020). Taekwondo anaerobic intermittent kick test: discriminant validity and an update with the gold-standard wingate test. J. Hum. Kinet. 71 (31), 229–242. 10.2478/hukin-2019-0081 32148587 PMC7052711

[B44] Tayech A. Mejri M. A. Makhlouf I. Uthoff A. Hambli M. Behm D. G. (2022). Reliability, criterion-concurrent validity, and construct-discriminant validity of a head-marking version of the taekwondo anaerobic intermittent kick test. Biol. Sport 39 (4), 951–963. 10.5114/biolsport.2022.109459 36247969 PMC9536368

[B45] Weir J. P. (2005). Quantifying test-retest reliability using the intraclass correlation coefficient and the SEM. J. Strength Cond. Res. 19 (1), 231–240. 10.1519/15184.1 15705040

[B46] World Medical Association (2013). World medical association declaration of helsinki: ethical principles for medical research involving human subjects. J. A. M. A 310 (20), 2191–2194. 10.1001/jama.2013.281053 24141714

